# *Yupingfeng* polysaccharide promote the growth of chickens via regulating gut microbiota

**DOI:** 10.3389/fvets.2024.1337698

**Published:** 2024-02-23

**Authors:** Yuling Guan, Wendan Zheng, Yu Bai, Bo Wu

**Affiliations:** Guangdong Provincial Key Laboratory of Animal Molecular Design and Precise Breeding, School of Life Science and Engineering, Foshan University, Foshan, China

**Keywords:** *Yupingfeng* polysaccharide, chicken, antibiotics, production performance, gut microbiota

## Abstract

**Introduction:**

*Yupingfeng* polysaccharide (YPF-P) is the main substance of alcohol deposition in *Yupingfeng* powder, which has many biological functions such as enhancing immunity, repairing intestinal barrier and enhancing antioxidant ability. This study employed *in vitro* growth-promoting drug feed additives and animal experiments to comprehensively evaluate the use of YPF-P in broiler production.

**Methods:**

A total of 1,296 151 days-old *Qingyuan* Partridge chickens were randomly divided into four groups with six replicates and 54 hens per replicate: the control group was fed basal diet, and the experimental groups were fed diets supplemented with 4 g/kg, 8 g/kg, and 12 g/kg YPF-P for 14 days. Broilers were weighed before and at the end of the experiment to calculate total weight gain (GW), average daily gain (ADG), and feed compensation. At the end of the experiment, six chickens from each group were randomly selected for subwing vein blood sampling, which was used to measure serum biochemical indicators GHRH, GH, and IGF-1 by ELISA method. Randomly select chickens from control group and 8 g/kg group for slaughter, and cecal contents were collected for 16S high-throughput sequencing.

**Results:**

Dietary supplementation of 8 g/kg YPF-P can significantly increase the final body weight, total weight gain, average daily gain and decrease the feed to gain ratio of chickens. During 151–165 days, serum IGF-1 concentrations increased significantly (*p* < 0.05). There were no significant changes in serum GH concentration (*p* > 0.05). In terms of gut microbiota, there was no significant difference between control group and test group in Shannon index and Simpson index. Compared with the control group,the addition of 8 g/kgYPF-P significantly increased the abundance of *Firmicutes* and significantly decreased the abundance of Bacteroides at the phylum level.At the genus level, the relative abundance of *unclassified_Oscillospiraceae* was significantly increased and the *unclassified_Muribaculaceae*, *uncultured_Bacteroidales_bacterium*, *Lactobacillus*, *Alloprevotella*, *Ligilactobacillus*, *Prevotellaceae_UCG_001*, and *unclassified_Atopobiaceae* was significantly decreased.

**Conclusion:**

The above results showed that adding 8 mg/kg of YPF-P could increase the average daily gain of *Qingyuan* Partridge chickens, reduce the ratio of feed to meat, and affect the distribution proportion of intestinal microflora in chickens to some extent.

## 1 Introduction

Antibiotics have bacteriostatic or bactericidal effects. Studies have shown that using appropriate doses of antibiotics in the feed of food animals can prevent animal diseases and improve production performance (1–3). This has been common practice in modern animal husbandry for decades. However, the irrational use of antibiotics has led to the emergence of more and more drug-resistant bacteria. These antibiotic-resistant bacteria remain in animals and are easily passed to humans through the food chain (4), and these antibiotic-resistant bacteria can be pathogenic to humans and widely spread in the environment through animal feces (5), thus affecting animal and human health and food safety. This has raised concerns, prompting efforts to develop so-called alternatives to antibiotics.

At present, the natural antibiotic substitutes found by people are mainly beneficial prebiotics, probiotics, alkaloids, antimicrobial peptides, essential oils, and plant substances, etc. (4, 6–10), the most common feed additives have been widely used in the animal husbandry industry after antibiotic growth promoters were banned. Plant-derived feed additives include herbs, plant extracts (essential oils, oil-based resins, tannins, saponins, polysaccharides, phenols, flavonoids, etc.), and their active ingredients (11–13). As feed additives, these substances can promote the growth of livestock and poultry, increase the feed reward, and improve the immune capacity and antioxidant capacity of the animal body. Olawuwo’s et al. research shows that (14), the powder of *Morinda lucida*, *Acalypha wilkesiana*, and *Ficus exasperata* is higher in macronutrients and micronutrients, such as K, Ca^2+^, Na^+^, Mg^2+^, and P, than the standard broiler feed. It is also rich in organic acids and sugars required for livestock nutrition and can effectively maintain antioxidant defenses. To improve animal health and promote the effect of growth performance. Studies have also shown that the addition of *Artemisia lordosica* polysaccharide can play an anti-inflammatory and antioxidant role, improve the protein digestibility of broilers, reduce the content of serum stress hormones and pro-inflammatory cytokines, and alleviate the growth inhibition and intestinal damage induced by lipopolysaccharides (LPS) (15). Dietary supplementation of *Origanum vulgare* and *Andrographis paniculata* extract (FOA) (16) can reduce feed intake, increase the ratio of beneficial and harmful bacteria in the gut, reduce coccidium oocysts and alleviate intestinal lesions of broilers. To sum up, the plants extract substances added can promote the growth of poultry and intestinal health, improve the body, and improve anti-inflammatory and antioxidant function.

Yupingfeng powder, a traditional Chinese medicine formula, is composed of *Astragalus membranaceus*, *Atractylodes macrocephala Koidz*. and *Saposhnikovia divaricata (Turcz.) Schischk* (17). It has the functions of immune regulation (18), anti-virus (19), anti-tumor (20), and anti-aging (21). Yupingfeng polysaccharide is the main substance in the alcohol-precipitation part of Yupingfeng powder, which has many biological functions such as enhancing the body’s immunity (22), repairing the intestinal barrier (23), and enhancing the body’s antioxidant capacity (24). For example, YPF-P significantly increased the thymus index and promoted the secretion of total antioxidant capacity (T-AOC), superoxide dismutase (SOD), glutathione peroxidase (GSH-Px), immunoglobulin A (IgA), immunoglobulin A (IgG) and immunoglobulin M (IgM) and other immune factors and antioxidant factors in the blood (25). The study by Chen et al. also showed that dietary Yupingfeng polysaccharide supplementation could significantly increase the expression of immune-related genes in blood cells and the intestinal tract; In addition, it can also reduce the cumulative mortality of *Litopenaeus vannamei* after *Vibrio harveyi* attack (26). The results showed that YPF-P is beneficial to animals, and can play a certain role in immune response, oxidative stress, and intestinal homeostasis of animals. YPF-P is a worthwhile green plant additive.

The gut is the largest body interface in contact with the environment (27), its primary function is to digest and absorb nutrients while forming a barrier against luminal pathogens such as antigenic peptides, bacteria, toxins, parasites, allergens and carcinogens (28, 29). The intestinal tract is rich in microorganisms [~10^12^–10^13^, (30)], mainly composed of anaerobic bacteria, and it is challenging to explore the microbial diversity of healthy intestinal flora using traditional culture methods (31). Guo et al. showed that *Lentinus edodes* extract (LenE), *Tremella fuciformis* extract, and *Astragalus membranaceus Radix* extract could increase the number of potentially beneficial bacteria (*Bifidobacterium* and *Lactobacillus*) in chicken cecum microflora while decreasing the number of potentially harmful bacteria (*Bacteroides* and *Escherichia coli*) (32), these polysaccharides extracts can be used as regulators of intestinal microorganisms in broilers to promote body health. More recently, natural plant polysaccharides, which are considered quality prebiotics, have been shown to reach the large intestine, where they can interact with the gut microbiota (GM) (33), where they can promote the proliferation of beneficial bacteria and inhibit the overgrowth and reproduction of foreign bacteria and potentially pathogenic bacteria, thereby regulating gut health (34). This study investigated the effects of different concentrations of *Yupingfeng* polysaccharide as a feed additive on growth performance and intestinal flora of *Qingyuan* Partridge chickens, aiming to provide the theoretical basis for *Yupingfeng* polysaccharide as a green and healthy feed additive for the poultry production industry.

## 2 Materials and methods

### 2.1 Drugs and reagents

*Yupingfeng* polysaccharide were provided by Foshan Dezhong Pharmaceutical Co., LTD. In the preparation process of *Yupingfeng* polysaccharide, Astragalus membranaceus, Atractylodes macrocephala Koidz. and Saposhnikovia divaricata (Turcz.) Schischk. were weighed according to the weight ratio of 3:1:1. The herbal mixture was boiled for 2 h in 8 volumes of water (v/w) and again for 1 h in 6 volumes of water. The two extracts were mixed and filtered, lyophilized and stored at 4°C.

### 2.2 Animals and experimental design

A total of 1,296 *Qingyuan* Partridge chickens (151 days-old, female) with similar initial body weight were randomly divided into four groups, with six replicates per group and 54 chickens per replicate, every three chickens are raised in a cage. *Qingyuan* Partridge chicken is raised in layer cage. During the whole test period, the temperature of the room in which the chickens were kept was maintained between 20°C and 26°C, with a light duration of 16 h per day and a light intensity of 20 to 30 lx. The diets were as follows: (1) control group, basal diet; (2) YC1, basal diet +4 g/kg YPF-P; (3) YC2, basal diet +8 g/kg YPF-P; (4) YC3, basal diet +12 g/kg YPF-P. The period of 65 days was carried out in the last 2 weeks before the Column quantity. *Qingyuan* Partridge chickens and standard feed used in this experiment were purchased from Guangdong Tiannong Food Co., LTD. (Guangdong, China). This experiment was approved by the Institutional Experimental Animal Ethics Committee of Foshan University (FOSU 2022-292).

The basic diets of this experiment are shown in Table 1. The *Yupingfeng* polysaccharide was provided in powder form and added to the base diet, and was fed once a day at an average of 100 g/piece at 8:00 am. During the experiment, the free drinking water of *Qingyuan* polysaccharide chicken was guaranteed. At the end of the experiment, a chicken with a body weight close to the average was selected from each replicate, weighed and recorded the weight of the broiler, and blood samples were collected from the subwing vein. Finally, the chickens were killed and the cecal contents were collected, and the above samples were sent to Beijing Baimaike Biotechnology Co., LTD for 16S sequencing.

**Table 1:** Ingredient and nutrient composition of basal diet (as feed).

Item	Composition
Ingredient (%)	
Corn	61.03
Soybean meal	30.80
Soybean oil	2.50
Fish meal	1.50
CaHPO4	1.40
Limestone	1.40
NaCl	0.37
Premix^a^	1.00
Total	100.00
Calculated nutrient levels (%)	
Crude protein	15.00
Calcium	0.64
Available phosphorus	0.41
Methionine	0.29
Methionine + cysteine	0.52
Metabolizable energy (MJ kg^−1^)	12.40

a The premix provided the following per kg of diet: vitamin A, 9500 IU; vitamin B1, 1.5 mg; vitamin B2, 9.0 mg; vitamin B6, 3.0 mg; vitamin B12, 0.02 mg; vitamin D3, 2,375 IU; vitamin E, 19 IU; vitamin K3, 1.40 mg; biotin, 0.95 mg; folic acid, 0.93 mg; D-pantothenic acid, 9.3 mg; Cu (as copper sulfate), 15 mg; Fe (as ferrous sulfate), 60 mg; Mn (as manganese sulfate), 100 mg, Zn (as zinc sulfate), 70 mg; I (as potassium iodide), 0.50 mg; Se (as sodium selenite), 0.59 mg.

### 2.3 Growth performance

The initial body weight (IW) of broilers was recorded before the start of the experiment. On the 14th day of the experiment, broilers were fasted for 12 h, then the body weight (FW) of broilers was measured, and the growth was measured based on the average weight gain (DW) of surviving broilers in each group. Average daily gain (ADG), average daily feed intake (ADFI), and feed conversion (ADFI/ADG) were calculated.


ADG=(FW−IW)/14d

### 2.4 Serum biochemical indicators

At the end of the experimental period, six chickens were randomly selected from each group (one bird per replicate). After fasting for 12 h (taking water at will), a blood sample was obtained from the broiler by puncturing the wing vein of the broiler and the sample was transferred to a 1.5 mL Eppendorf tube. The tubes were centrifuged at 4°C at 3,000 × g for 15 min to isolate the serum, which was then stored at −20°C for analysis of serum parameters. According to the manufacturer’s instructions, the contents of growth hormone (GH) and insulin-like growth factor-1 (IGF-1) were determined by ELISA kit (Shanghai enzyme-linked Biotechnology Co., Ltd., Shanghai, China).

### 2.5 16 s rRNA sequencing analysis of cecal microorganisms

Cecal contents isolated after slaughter. Total genomic DNA was extracted from Cecal contents samples using the TGuide S96 Magnetic Soil/Stool DNA Kit Tiangen Biotech (Beijing Co., Ltd.) according to the manufacturer’s instructions. The hypervariable region V3–V4 of the bacterial 16S rRNA gene were amplified with primer pairs 338F: 5′-ACTCCTACGGGAGGCAGCA-3′ and 806R: 5′-GGACTACHVGGGTWTCTAAT-3′. PCR products were checked on agarose gel and purified through the Omega DNA purification kit (Omega Inc., Norcross, GA, United States). The purified PCR products were collected and the paired ends (2 × 250 bp) were performed on the Illumina Novaseq 6,000 platform.

The qualified sequences with more than 97% similarity thresholds were allocated to one operational taxonomic unit (OTU) using USEARCH (version 10.0). Taxonomy annotation of the OTUs/ASVs was performed based on the Naive Bayes classifier in QIIME2 using the SILVA database (35) (release 138.1) with a confidence threshold of 70%. Alpha was performed to identify the complexity of species diversity of each sample utilizing QIIME2 software. Beta diversity calculations were analyzed by principal coordinate analysis (PCoA) to assess the diversity in samples for species complexity. One-way analysis of variance was used to compare bacterial abundance and diversity. Linear discriminant analysis (LDA) coupled with effect size (LEfSe) was applied to evaluate the differentially abundant taxa. The online platform BMKCloud^1^ was used to analyze the sequencing data.

### 2.6 Statistical analysis

SPSS statistical software (Version 27 for windows, SPSS Inc., Chicago, IL, United States) was used for one-way analysis of variance (ANOVA). Duncan’s multirange test was used to examine the differences between treatments. All results are expressed in mean and standard error (SEM) of mean unless otherwise stated, *p* < 0.05 was considered statistically significant.

## 3 Results

### 3.1 Effect of *Yupingfeng* polysaccharide on growth performance of *Qingyuan* Partridge chickens

At the end of the experiment, compared with the control group, the final body weight, total weight gain and average daily gain of 8 g/kg *Qingyuan* Partridge chickens were significantly higher (*p* < 0.05), while the feed-meat ratio was significantly lower (*p* < 0.05) (see Table 2).

**Table 2:** Effects of adding different concentrations of *Yupingfeng* polysaccharide on production performance of *Qingyuan* Partridge chicken.

	The added amount of *Yupingfeng* polysaccharide (g/kg)			
Item	0.00	4.00	8.00	12.00
IW(kg)	1.77 ± 0.01	1.77 ± 0.01	1.78 ± 0.01	1.77 ± 0.01
FW(kg)	1.78 ± 0.02^b^	1.80 ± 0.02^b^	1.89 ± 0.03^a^	1.81 ± 0.02^ab^
GW(g)	20.74 ± 13.99^b^	39.07 ± 11.18^ab^	68.91 ± 10.18^a^	58.79 ± 12.06^ab^
ADG(g)	1.54 ± 1.04^b^	2.79 ± 0.80^ab^	4.92 ± 0.73^a^	4.20 ± 0.86^ab^
F/G(g:g)	17.41 ± 1.37^a^	15.17 ± 1.32^ab^	13.04 ± 0.67^b^	14.35 ± 1.21^ab^

The average value of different shoulder label letters in the same row indicates significant difference (*p* < 0.05). IW, initial weight; FW, weight at the end of the trial; GW, weight gain during the test; ADG, average daily gain; F/G, feed to weight gain ratio.

### 3.2 Effect of *Yupingfeng* polysaccharide on serum parameters of *Qingyuan* Partridge chickens

**Figure 1:**
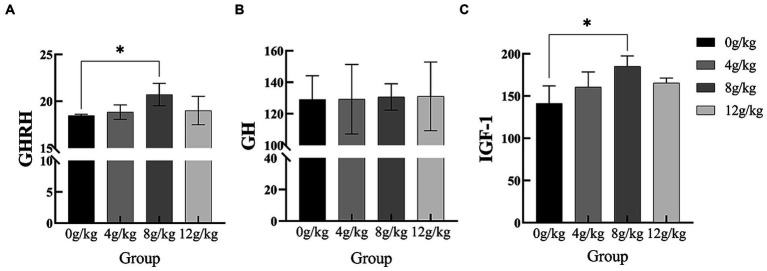
Effect of *Yupingfeng* polysaccharide on serum parameters of *Qingyuan* Partridge chickens. **(A)** Growth hormone releasing hormone (GHRH); **(B)** Growth hormone (GH); **(C)** Insulin-like growth factor-1 (IGF-1).

As shown in Figure 1, there were significant increases in serum GHRH and IGF-1 concentrations during 151–165d (*p* < 0.05). In addition, YPF-P supplementation did not affect serum GH concentration (*p* > 0.05).

### 3.3 Effect of *Yupingfeng* polysaccharide on intestinal flora of *Qingyuan* Partridge chickens

#### 3.3.1 Sequence and out data

**Figure 2:**
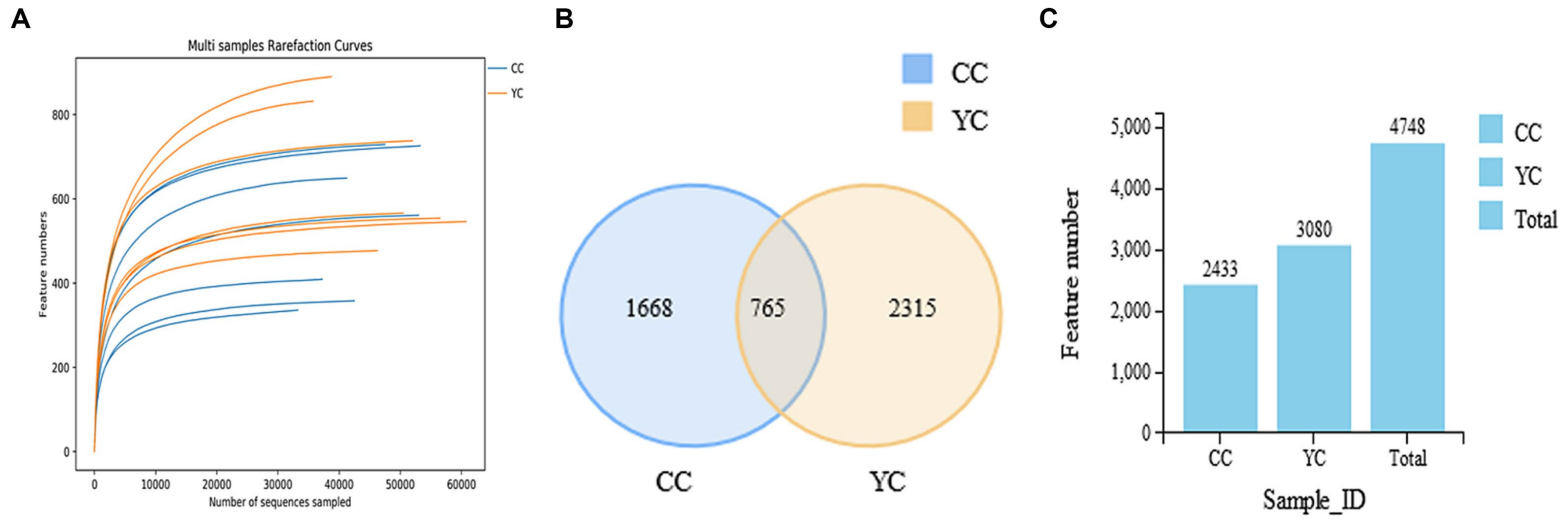
Dilution curve, number of OUT distributions and Venn diagram. **(A)** Dilutive curve of gut microbes. Abscissa: number of sequencing data; Ordinate: number of species observed. **(B)** Two groups of OTU distribution histogram; **(C)** Veen. CC, control group; YPF-P, supplemental level was 0 g/kg; YC, YPF-P supplemental amount is 8 g/kg.

The original data was spliced (FLASH, version 1.2.11), the spliced sequences were qualitatively filtered (Trimmomatic, version 0.33), and the illusion was removed (UCHIME, version 8.1) to obtain high-quality Tags sequences. The collected data were analyzed, and draw the dilution curve (Figure 2A). The curve is asymptotic to the *X*-axis, indicating that the species in this environment will not increase significantly with the number of sequenced, and it is close to saturation. Sequences were clustered at the similarity level of 97% (USEARCH, version 10.0), and 0.005% of all sequences sequenced were filtered as a threshold. The filtered sequences became outs, and similar OTUs were intersected in the Venn diagram. Results as shown in Figures 2B,C, a total of 4,748 outs were aggregated in the two groups, of which 1,688 were unique to the control group, and 2,315 were unique to the test group.

#### 3.3.2 Effects of *Yupingfeng* polysaccharide on intestinal flora diversity of *Qingyuan* Partridge chickens

**Figure 3:**
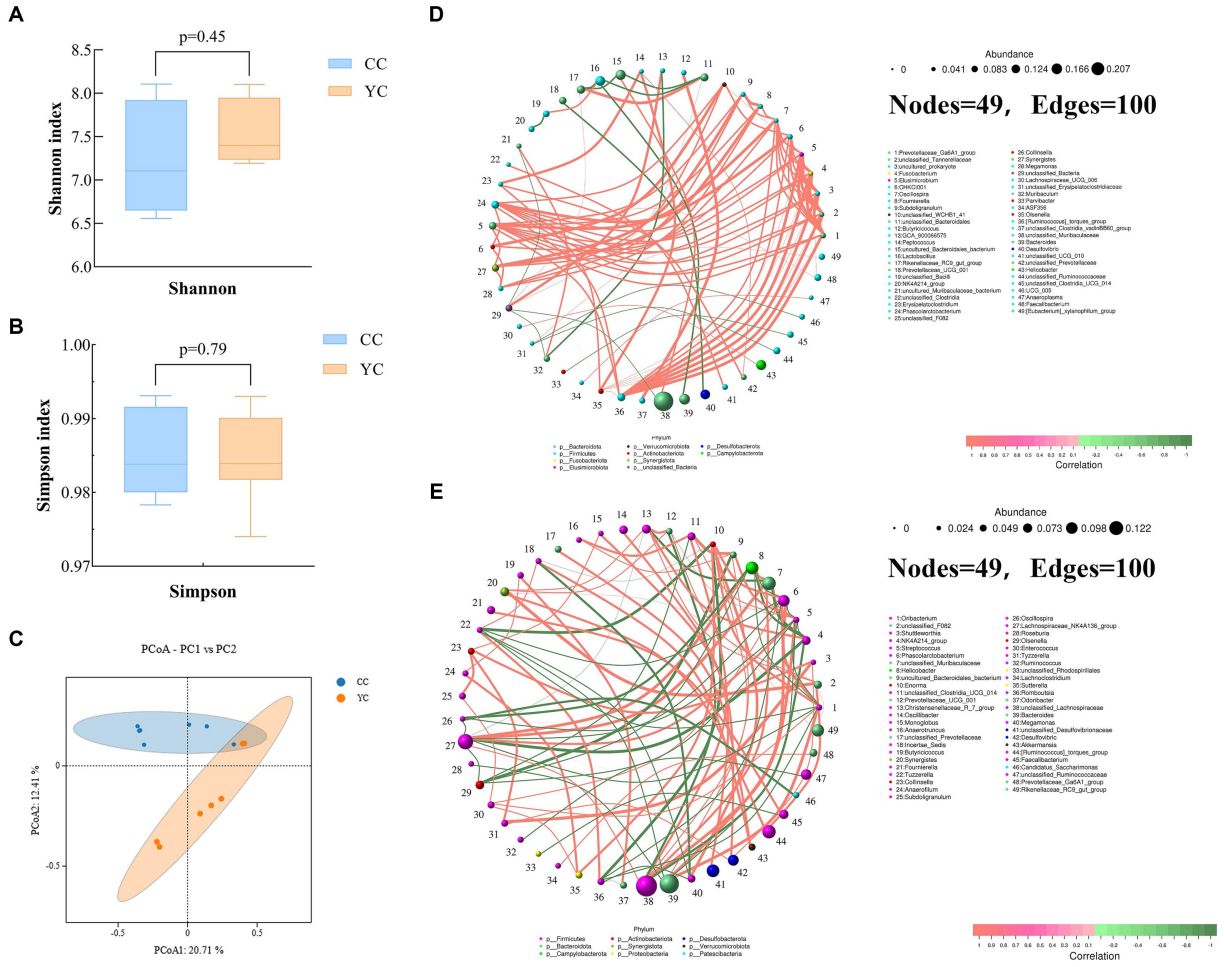
Effects of *Yupingfeng* polysaccharide on intestinal flora diversity of *Qingyuan* Partridge chickens. **(A)** The Simpson index results of alpha diversity of cecal microbiota in different groups. Data were analyzed by Student’s *t* test; **(B)** The Simpson index results of alpha diversity of cecal microbiota in different groups. Data were analyzed by Student’s *t* test; **(C)** Principal coordinate analysis (PCoA) of cecal microbiota in different groups. PCoA plotswere generated using OUT abundance data according to the Binary–Jaccard distance algorithm; **(D)** Correlation network analysis of cecal microorganisms based on core genera of CC group. Different colors of nodes indicate different phylum levels. Edges represent significant correlations (*p* < 0.05); **(E)** Correlation network analysis of cecal microorganisms based on core genera of YC group. Different colors of nodes indicate differentphylum levels. Edges represent significant correlations (*p* < 0.05).

Shannon index and Simpson index were used to measure the *α* diversity of species, and the *β* diversity of cecal flora between different groups was displayed by principal coordinate analysis (PCoA) results. The results of cecal microbial α diversity analysis between the two groups are shown in Figures 3A,B. Compared with the control group, there were no significant differences in Shannon index and Simpson index in YC polysaccharide addition group (YC group). However, all of them were higher than those in the control group, indicating that the addition of polysaccharide had a tendency to increase the diversity of cecal flora. The results of PCoA analysis (Figure 3C) showed that the microbial communities of the control group and the *Yupingfeng* polysaccharide addition group were significantly separated, and the contribution rates of the first two principal components PCoA1 and PCoA2 were 20.71% and 12.41%, respectively, which revealed certain changes in intestinal flora. Correlation network analysis based on relative abundance at the genus level showed that both CC group and YC group had 49 nodes and 100 edges, but the core genera of CC group and YC group contained 10 phylum and 9 phylum, respectively, indicating that YPF-P could change the distribution of cecal microflora of *Qingyuan* Partridge Chicken (Figures 3D,E).

**Figure 4:**
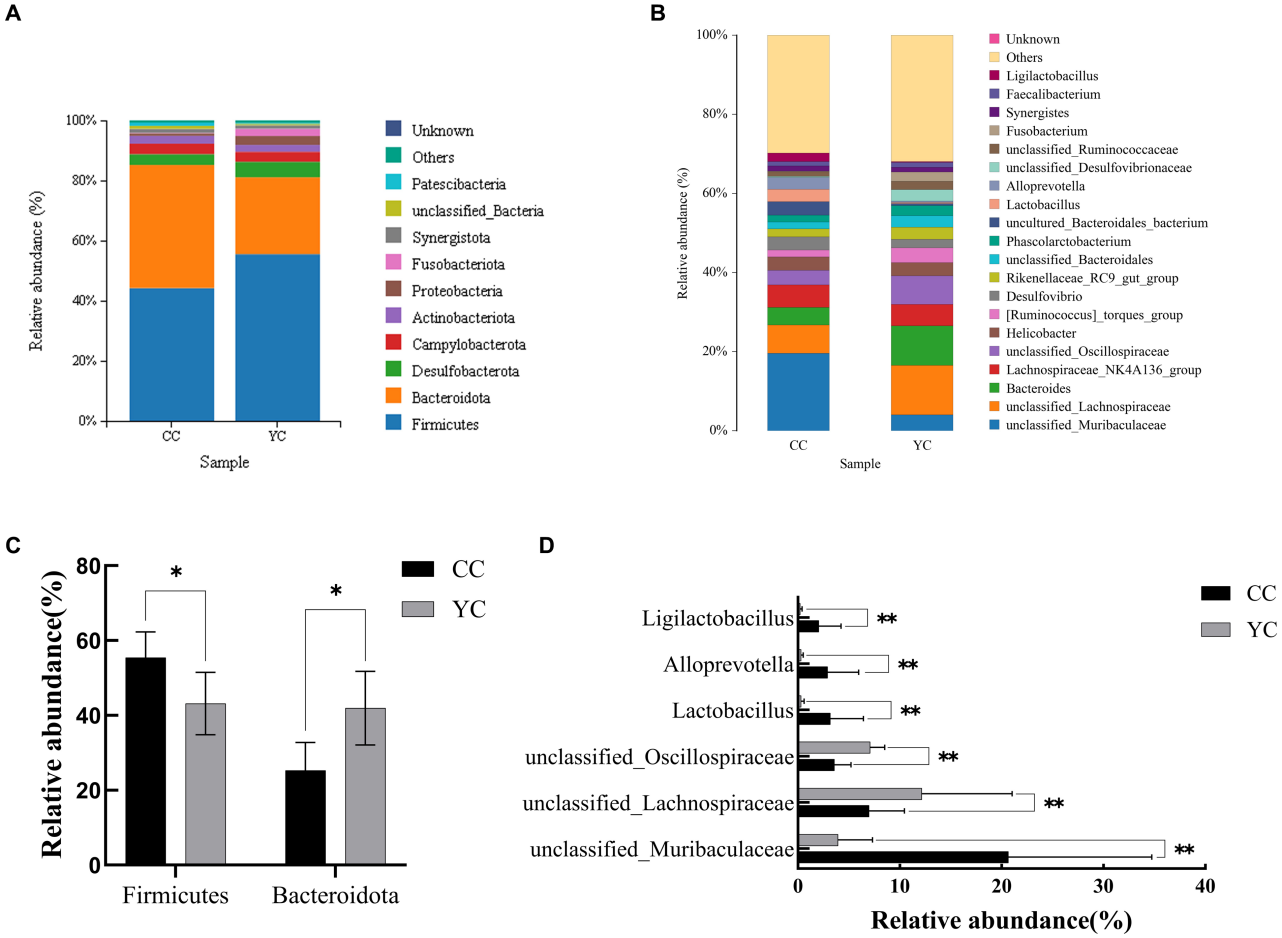
Effects of *Yupingfeng* polysaccharide on intestinal microflora phylum and genus level of *Qingyuan* Partridge chickens. **(A)** Phylum-level species distribution bar chart, showing only the top 10 species; **(B)** Genus level species distribution bar chart, showing only the top 20 species; **(C)** Bacterialphyla with significant differences at phylum level; **(D)** Bacterial genera with significant differences at the genus level.

Using SILVA as a reference database, the naive Bayes classifier was used to annotate the feature sequences, and the species classification information corresponding to each feature could be obtained. Then the sample community composition could be counted at various levels (phylum, class, order, family, genus, species). QIIME software was used to generate species abundance tables at different taxonomic levels, and R were used to draw community structure histogram at different taxonomic levels. Species distribution histogram at phylum level and genus level was selected to display the results, as shown in Figure 4. The analysis of species annotations at the phylum classification level (Figure 4A) showed that the top 10 intestinal flora included *Firmicutes*, *Bacteroideta*, *Desulfobacterota*, *Campylobacterota*, *Actinobacteriota*, *Proteobacteria*, *Fusobacteriota*, and *Synergistota*, among which Firmicutes and Bacteroidetes accounted for the highest content, which could reach more than 80% of the relative abundance. There were *Firmicutes* (43.18%) and *Bacteroidetes* (41.94%) in the control group and *Firmicutes* (55.45%) and *Bacteroidetes* (25.30%) in the YC group. Compared with the control group, the *Firmicutes* were significantly increased (*p* < 0.05) and *Bacteroidetes* were significantly decreased (*p* < 0.05) in the YC group (Figure 4C). The species annotation analysis at the genus classification level (Figure 4B) showed that the dominant flora were: *unclassified_Muribaculaceae*, *unclassified_Lachnospiraceae*, *Bacteroides*, *Lachnospiraceae_NK4A136_group*, *unclassified_Oscillospiraceae*, *Helicobacter*, *[Ruminococcus]_torques__group*, *Desulfovibrio*, *Rikenellaceae_Rc9_gut_group*, *unclassified_Bacteroidales*, et al. Among them, there was a significant difference between the two groups in *unclassified_Muribaculaceae*, *uncultured_Bacteroidales_bacterium*, *unclassified_Oscillospiraceae*, *Lactobacillus*, *Alloprevotella*, *Ligilactobacillus*, *Prevotellaceae_UCG_001*, and *unclassified_Atopobiaceae* (Figure 4D), then *unclassified_Oscillospiraceae* was significantly increased, while several other differential genera were significantly decreased.

#### 3.3.3 Functional predictive analysis

**Figure 5:**
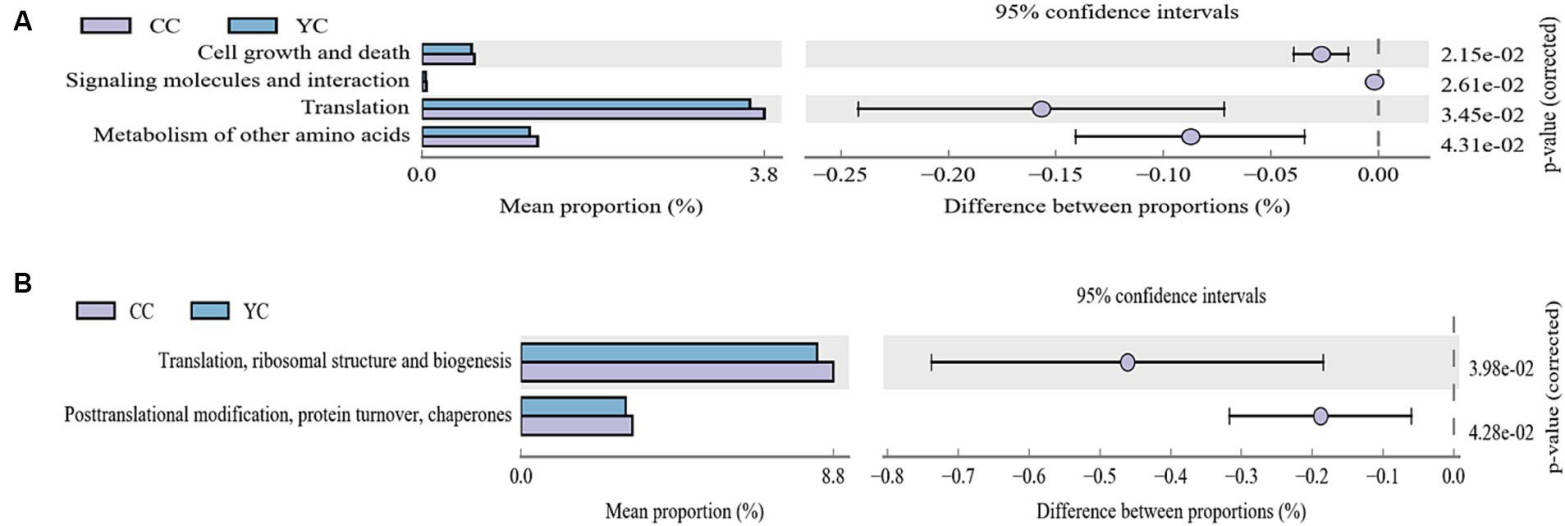
Functional predictive analysis. **(A)** Difference analysis diagram of KEGG metabolic pathway at the second level. Different colors in the diagram represent different groups. **(B)** Statistical results of COG functional classification in the second level. The figure on the left shows the abundance ratio of different functions in two samples or two groups of samples, the middle shows the difference ratio of functional abundance in the 95% confidence interval, and the value on the far right is the *p*-value.

Picrust2(2.3.0) software was used to compare the sample 16SrDNA with the reference sequence of the Integrated Microbial Genome (IMG) database, build the species evolutionary tree, and identify the closest species. Corresponding to the family information in KEGG and COG databases, the analysis results of KEGG metabolic pathway differences between groups and the statistical results of COG functional classification are shown in Figure 5. Figure 5A shows differences in metabolic pathways between control and experimental groups, including cell growth and death, signaling molecules and interactions interaction, translation, and metabolism of other amino acids pathway. Functional differences between the two groups were translation, ribosomal structure and biogenesis, post-translation modification, protein turnover, and chaperones (Figure 5B).

## 4 Discussion

In recent years, studies have shown that the extracts of medicinal plant active ingredients, including Chinese herbal polysaccharides, have been playing a positive role in livestock production and disease prevention and treatment (12). Chinese herbal polysaccharide can play a variety of biological functions (36, 37), such as anti-inflammatory (38), anti-tumor (39), antioxidant, lowering blood sugar, lowering blood lipids (40), immune regulation (41), and regulating intestinal flora (42), etc. Chen et al. found that the basal diet supplemented with 1,000 mg/kg *Lonicera fulvotomentosa* extract could significantly increase the final weight of weaned piglets and the diversity and richness of intestinal flora in the jejunum and rectum. With the increase inf extract concentration (600 mg/kg, 800 mg/kg, 1,000 mg/kg), the diarrhea index of piglets was decreased [*p* < 0.05, (43)]. Appropriate doses of *Piper betel* (PB) (4 g/kg) and *Persicaria odorata* (PO) (8 g/kg) can also be used as growth promoters to significantly increase weight gain and reduce feed reward in broilers while positively regulating intestinal structure (44). Reda observed that adding 750 and 1,000 mg/kg of *Licorice* (*Glycyrrhiza glabra*) to the diet of Japanese quails enhanced the growth performance of the animals and reduced intestinal pathogens (45). These results are consistent with our experimental results. In this experiment, when the added amount of *Yupingfeng* polysaccharide is 8 g/kg, the final body weight, the total weight gain and the average daily gain of *Qingyuan* Partridge chickens can be significantly improved (*p* < 0.05), and the feed-to-gain ratio can be significantly reduced (*p* < 0.05). Therefore, appropriate concentration added to the basic diet of jade screen polysaccharide can improve chicken growth performance.

Growth hormone and insulin-like growth factor are both peptides that can promote growth and play an active role in the body’s growth, development and metabolism. Growth hormone, also known as somatotropin, has widely distributed receptors that stimulate the growth and differentiation of muscle, bone, and cartilage. On the other hand, growth hormone also stimulates the production of insulin-like growth factors (IGFs), which can indirectly mediate many of the growth-promoting effects of growth hormone (46). There are two forms of IGF, IGF-I and IGF-II. Among them, the production of IGF-I is more dependent on GH, and its growth-promoting effect is stronger. Some studies have shown that dietary supplementation of *Radix Rehmanniae Preparata* polysaccharides (RRPP) can promote and significantly improve growth performance (*p* < 0.05); the mRNA expression levels of serum growth hormone (GH), insulin-like growth factor IGF-I and IGF-II were significantly increased by RRPP supplementation (47). Similarly, the research of Zhao et al. (48) also has the same conclusion: in early weaned piglets, diets supplemented with different concentrations of *Mulberry leaf* polysaccharides (MLPs) and antibiotics found that IGF-I and GH levels in *Mulberry leaf* polysaccharide treated (MT) group were significantly higher than those in control treated (CT) group and antibiotic treated (AT) group (*p* < 0.05). Ouyang et al. (49) also reported that supplementation with 1.0% and 1.5% Ouyang et al. also reported that supplementation with 1.0% and 1.5% *water soluble Alfalfa* olysaccharides (WSAP) significantly or extremely significantly increased the expression of GH and IGF-I genes in liver, adipose tissue and kidney of broilers (*p* < 0.05 or *p* < 0.01), and improved the growth performance and carcass traits of broilers. In this experiment, the same conclusion was also obtained, when the added amount of YPF-P was 8 g/kg, the expression levels of IGF-I were significantly increased (*p* < 0.05), and the concentration of GH was not significantly changed, but there was a trend of increase. This is also consistent with our results in the previous part, when the amount of *Yupingfeng* polysaccharide added to the diet was 8 g/kg, the weight gain of *Qingyuan* Partridge chicken was the most obvious, and the feed reward was the lowest. Therefore, it was concluded that *Yupingfeng* polysaccharide could promote growth.

A large microbial community exists in the gastrointestinal tract and these microorganisms play an important role in the growth and health of chickens, promoting the absorption of nutrients and improving the immune system (50). The addition of Chinese herbal ingredients and their derivatives can improve the community composition of intestinal microorganisms of broilers, affect the digestion and absorption of nutrients, and thus affect the growth performance and immune performance of broilers (51–56). *α* diversity results showed that compared with the control group, there were no significant differences in Shannon, and Simpson indices in the YC group, indicating that adding *Yupingfeng* polysaccharide did not change the species diversity and richness of intestinal flora. The PCoA map of *β* diversity results showed that the intestinal flora of the control group and the experimental group were significantly separated, with little overlap, and there was a significant difference between the groups, suggesting that there was a substantial difference in the microbial community between the two groups, and the analysis results were reliable. At the phylum level, *Firmicutes*, *Bacteroidetes*, *Desulfobacterota*, *Campylobacter*, *Actinobacteria*, and *Proteobacteria* were the main microbial groups in the cecum, among which *Firmicutes* (43.18%) and *Bacteroidetes* (41.94%) were in the control group, and *Firmicutes* (55.45%) and *Bacteroidetes* (25.30%) were in the YC group. Studies by Li et al. showed that *Bacteroides*, *Firmicutes*, *Proteus*, and *Ferribacter*, accounted for more than 97% of the cecal microbiota of all chickens (57); the top two are *Firmicutes* and *Bacteroidetes*, which are consistent with our results. Hou et al. found that in Arbor Acres broilers fed the same diet in FL and LL lines, Firmicutes and Bacteroides accounted for 71.36% and 23.40% of the intestinal flora in fat chickens, 53.44% and 41.09% in lean chickens, respectively (58). Consistent with the findings of *Furet* and *Chika Kasai*, non-obese subjects had higher *Bacteroides* abundances at the phylum level in their gut microbes compared to obese subjects (59, 60). In this experiment, the addition of *Yupingfeng* polysaccharide tended to reduce the relative abundance of *Bacteroidetes*, consistent with the study’s results, and the ratio of *Firmicutes* to *Bacteroidetes* increased, and the weight of birds increased. At the genus level, the predominant bacteria groups of cecum contents are *unclassified_Muribaculaceae*, *unclassified_Lachnospiraceae*, *Bacteroides*, *Lachnospiraceae_NK4A136_group*, and *unclassified_Oscillospiraceae*. Among them, the first three belong to Bacteroides, while the last two belong to *Firmicutes* and *Clostridium*. The flora that differed between the CC and YC group were *unclassified_Muribaculaceaeand unclassified_Oscillospiraceae*. Hu et al. found that *Grifola frondosa powder suspension* (GFPS, powder of a medicinal fungus with anti-obesity properties) can reduce body weight and increase the abundance of *unclassified_Muribaculaceae* in the intestinal flora of mice (61), which is exactly consistent with our results. *unclassified_Muribaculaceae* is a bacterium that produces short-chain fatty acids, which are important in regulating glucose and lipid metabolism. These results indicate that *Yupingfeng* polysaccharide can regulate the abundance of different intestinal flora by increasing the proportion of beneficial bacteria and reducing the proportion of harmful bacteria to promote the growth performance of chickens.

## 5 Conclusion

In summary, this study shows that adding *Yupingfeng* polysaccharide to the basic diet can improve the growth performance of broilers and have a particular effect on the composition of their intestinal flora. When 8 g/kg *Yupingfeng* polysaccharide was added to the diet, the final body weight, the total weight gain and the average daily gain of *Qingyuan* Partridge chickens was increased (*p* < 0.05), and the feed reward was also decreased (*p* < 0.05). At the same time, it can promote the expression of GH and IGF-I in serum. Although adding *Yupingfeng* polysaccharide did not change the diversity and richness of intestinal flora, it could significantly affect the distribution proportion of dominant flora and increase the proportion of dominant flora. This study can provide the basic theoretical basis for utilizing *Yupingfeng* polysaccharide in broiler production.

## Data availability statement

The original contributions presented in the study are publicly available. This data can be found at: https://www.ncbi.nlm.nih.gov/bioproject/; PRJNA1067511.

## Ethics statement

The animal studies were approved by Institutional Experimental Animal Ethics Committee of Foshan University. The studies were conducted in accordance with the local legislation and institutional requirements. Written informed consent was obtained from the owners for the participation of their animals in this study.

## Author contributions

YG: Data curation, Formal analysis, Methodology, Writing – original draft. WZ: Methodology, Visualization, Writing – review & editing. YB: Data curation, Writing – review & editing. BW: Conceptualization, Methodology, Project administration, Supervision, Writing – review & editing.

## Footnotes

^1^ https://www.biocloud.net
